# “Real-world” radiomics from multi-vendor MRI: an original retrospective study on the prediction of nodal status and disease survival in breast cancer, as an exemplar to promote discussion of the wider issues

**DOI:** 10.1186/s40644-021-00406-6

**Published:** 2021-05-20

**Authors:** Simon J. Doran, Santosh Kumar, Matthew Orton, James d’Arcy, Fenna Kwaks, Elizabeth O’Flynn, Zaki Ahmed, Kate Downey, Mitch Dowsett, Nicholas Turner, Christina Messiou, Dow-Mu Koh

**Affiliations:** 1 Division of Radiotherapy and Imaging, Institute of Cancer Research, London, UK; 2 Department of Radiology, St George’s Hospital, London, UK; 3 Department of Radiology, Royal Marsden Hospital, London, UK; 4 Centre for Molecular Pathology, Royal Marsden Hospital, London, UK; 5 Breast Cancer Now Toby Robins Research Centre, The Institute of Cancer Research, London, UK; 6 Ralph Lauren Centre for Breast Cancer Research, Royal Marsden Hospital, London, UK

**Keywords:** Radiomics, Nodal status, Survival, Multi-vendor, Feature reduction

## Abstract

**Background:**

Most MRI radiomics studies to date, even multi-centre ones, have used “pure” datasets deliberately accrued from single-vendor, single-field-strength scanners. This does not reflect aspirations for the ultimate generalisability of AI models. We therefore investigated the development of a radiomics signature from heterogeneous data originating on six different imaging platforms, for a breast cancer exemplar, in order to provide input into future discussions of the viability of radiomics in “real-world” scenarios where image data are not controlled by specific trial protocols but reflective of routine clinical practice.

**Methods:**

One hundred fifty-six patients with pathologically proven breast cancer underwent multi-contrast MRI prior to neoadjuvant chemotherapy and/or surgery. From these, 92 patients were identified for whom T2-weighted, diffusion-weighted and contrast-enhanced T1-weighted sequences were available, as well as key clinicopathological variables. Regions-of-interest were drawn on the above image types and, from these, semantic and calculated radiomics features were derived. Classification models using a variety of methods, both with and without recursive feature elimination, were developed to predict pathological nodal status. Separately, we applied the same methods to analyse the information carried by the radiomic features regarding the originating scanner type and field strength. Repeated, ten-fold cross-validation was employed to verify the results. In parallel work, survival modelling was performed using random survival forests.

**Results:**

Prediction of nodal status yielded mean cross-validated AUC values of 0.735 ± 0.15 (SD) for clinical variables alone, 0.673 ± 0.16 (SD) for radiomic features only, and 0.764 ± 0.16 (SD) for radiomics and clinical features together. Prediction of scanner platform from the radiomics features yielded extremely high values of AUC between 0.91 and 1 for the different classes examined indicating the presence of confounding features for the nodal status classification task. Survival analysis, gave out-of-bag prediction errors of 19.3% (clinical features only), 36.9–51.8% (radiomic features from different combinations of image contrasts), and 26.7–35.6% (clinical plus radiomics features).

**Conclusions:**

Radiomic classification models whose predictive ability was consistent with previous single-vendor, single-field strength studies have been obtained from multi-vendor, multi-field-strength data, despite clear confounding information being present. However, our sample size was too small to obtain useful survival modelling results.

**Supplementary Information:**

The online version contains supplementary material available at 10.1186/s40644-021-00406-6.

## Introduction

Radiomics is a branch of image analysis that aims to extract quantitative features from radiographic images (including CT, MRI and PET) that are potentially beyond the perception of the human eye, in order to uncover novel features associated with treatment outcomes, disease molecular expressions or patient survival. Although radiomics approaches have been investigated for about a decade, the methodology is now being refined to ensure the derived signatures are robust, repeatable and meaningful [1, 2].

Many researchers (e.g., [3]) believe that image acquisition factors should be carefully controlled when used with radiomic analyses, especially in MRI, so as to prevent spurious findings, by avoiding artefactual clustering that may result from differences in scanner properties and/or acquisition techniques. Among the few multi-vendor radiomics studies with a clinical endpoint (as opposed to studies concentrating primarily on “feature reproducibility”), Mes et al. [4] were broadly encouraging, whereas Starmans et al. [5] observed a lack of translatability between vendors. In a recent review of harmonisation strategies, Da-Ano and colleagues make the point that “in MRI … [standardisation] guidelines are non-existent at the moment’” [6].

This concern can significantly limit the number of datasets that may be available for analysis, and also makes it difficult to compare results derived from different scanners both within and between imaging centres. Analysis is data-driven and there is no a priori way of determining how successful a given machine-learning model will be if provided with input data whose properties differ from those of the training data. Equally, a priori estimation of the significance of potential confounds such as field strength, acquisition acceleration via methodologies like SENSE [7] or compressed sensing [8], AI-based reconstruction strategies [9], or other vendor-specific post-processing appears impossible.

Furthermore, there is currently great interest in the development of AI models using data originating outside of clinical trials and hence without common acquisition protocols. There is thus a need for studies that assess the performance of radiomic methodologies using more heterogeneous, standard-of-care data: what we have termed “real-world radiomics”.

We choose as a case study the detection of lymph node disease originating from primary breast cancers. Nodal disease is an independent predictor for disease outcomes in patients with breast cancer, but definitive diagnosis is reliant on pathological examination of lymph nodes after surgery or via invasive lymph node tissue sampling. This is because imaging assessment based on nodal size measurement and/or morphological criteria has limited accuracy, and apical nodes in the axilla are poorly visualised by ultrasound at the time of diagnosis. Accurate pre-operative nodal staging can help to direct management by identifying those node-positive patients who would benefit from axillary nodal dissection, while at the same time prevent unnecessary morbidity associated with axillary clearance in those who are node-negative.

In the published literature to date [10–21] radiomics has been found to have moderate to good diagnostic accuracy (AUC 0.60–0.90) for determining nodal status in patients with breast cancer. In some studies [12, 16, 20], prediction using a combination of radiomics features and clinical risk factors led to further improvement in the identification of nodal status. However, these results have been derived from relatively “pure” study designs, where the MR images were sourced from specific scanner systems and a single field strength. This may limit their wider applicability and generalisability.

The hypothesis investigated here is that a predictive radiomics signal of disease status is detectable from clinical MRI images derived using analogous but not identical acquisition techniques across different scanner types, and that radiomics models developed in this way can overcome even gross confounds arising from differences in acquisition methodology. Hence, the aim of this study is to determine the real-world performance of MRI radiomics in patients with breast cancer to predict nodal disease status and patient disease survival and inform on the wider debate concerning best trial design for validating AI approaches in radiology.

## Materials and methods

### General methods

#### Patients

This was a retrospective study, which was approved by our institutional review board. Using the hospital electronic patient record, we identified patients diagnosed with breast cancer between 2007 and 2015. This period was chosen because the MRI scanners installed in our institution during this period were each in use for at least 5 years, and five-year cancer-survival follow-up data were available for patients.

#### Inclusion criteria

**Fig. 1:**
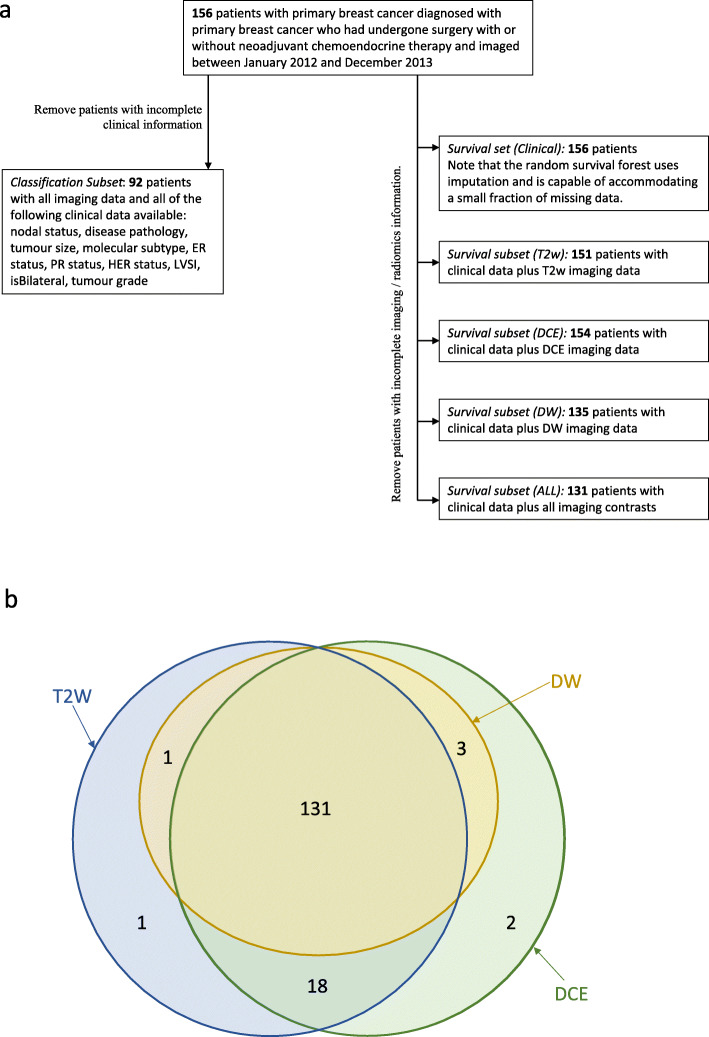
(**a**) Flow diagram of subject exclusion process; (**b**) Venn diagram illustrating availability of data between image contrast types and explaining the patient numbers in the right-hand side of (**a**)

(1) patients with pathologically proven breast cancer on whom a diagnostic breast MRI performed (*n* = 297) before surgery or neoadjuvant chemotherapy; (2) a mass lesion of at least 2 cm visible on MRI (*n* = 156) — this size threshold was chosen to ensure all ROIs would contain enough image pixels to calculate meaningful image features; (3) patients for whom imaging data in all three imaging contrasts (see below) were available, together with all of the key clinical and histopathological data listed in Fig. 1 (*n* = 92).

#### Exclusion criterion

significant artefacts visible on the breast MRI that precluded image analysis (*n* = 0).

All 156 patients (54 ± 12.5 years, IQR 16.3) were contoured as described below. Figure 1a shows a flow-chart of the stages of exclusion that resulted in the patient numbers quoted in the remainder of the article. Figure 1b is a Venn diagram showing the overlap of the available different contrast types in the imaging data.

#### Scanner models

Our cohort was deliberately heterogeneous in terms of the scanner model used and imaging magnetic field strength, with a view to testing the potential of radiomics in a “real-world” scenario. The scanner types and number of cases from each were: Siemens Aera 1.5 T (65), Siemens Avanto 1.5 T (14), Siemens Skyra 3 T (15), Philips Intera 1.5 T (1), Philips Achieva 1.5 T (32), Philips Achieva 3 T (29).

#### Image contrast types and sequences

Three different image contrasts were included in the study: T2-weighted, early-phase dynamic contrast-enhanced (subtraction images), and diffusion-weighted. The pulse sequences employed, patient procedures and contrast injection procedure were standard-of-care at the times of data acquisition, and the ranges of sequence parameter values are listed in Table 1 for each image contrast.

**Table 1:** Sequence parameters for the image data analysed

**MR Scanner**	
Manufacturer	Siemens, Philips
Model	Aera 1.5 T (65), Avanto 1.5 T (14), Intera 1.5 T (1), Achieva 1.5 T (32), Achieva 3 T (29), Skyra 3 T (15)
Field strength	1.5 T (112), 3 T (44)
**T2-weighted images**	
Sequence type	Multi-slice, turbo spin-echo, transverse
TR	3400–8690 ms
TE	70–120 ms
Matrix size x	448–576
Matrix size y	448–576
Matrix size z (number of slices)	34–60
Slice thickness	3.0–4.0 mm
**Dynamic contrast enhanced images**	
Contrast agent Typical administered dose	Dotarem® (Gadoteric acid - Gadoterate meglumine). 0.2 ml/kg
Typical injection rate	2–3 ml/s
Sequence type	3-D, T1-w subtraction
TR	3.9–5.5 ms
TE	1.5–2.6 ms
Flip angle	14–18°
Matrix size x	290–512
Matrix size y	290–512
Matrix size z	125–224
Slice thickness	1.0–2.5 mm
**Diffusion-weighted images**	
Sequence type	Diffusion-weighted multi-slice EPI, transverse
TR	2000–12,800 ms
TE	56–86 ms
Matrix size x	128–512
Matrix size y	72–404
Matrix size z	30–200
Slice thickness	3.0–5.0 mm

#### Data curation

Image data were sent from the Royal Marsden NHS Trust PACS to a local research PACS (based on the eXtensible Neuroimaging Archive Toolkit (XNAT) platform [22]), located inside the hospital’s clinical firewall and approved to hold identifiable patient data. Here, the data were pseudonymised and forwarded to a second XNAT instance outside the firewall which served as the primary data repository used for image analysis.

#### Image annotation

An annotation template was created using the AIM (Annotation and Imaging Markup) Template Builder [23] to capture image regions-of-interest (ROIs) and 12 radiologist-assessed semantic features (Table 2) based on MR BI-RADS descriptors. Images were initially viewed using ePAD (Rubin Lab, Stanford [24]), which also rendered the annotation template in the user interface.

**Table 2:** Radiologist semantic features captured

**T2-weighted images**	
Tumour visible (y / n)	
T2 signal (high / intermediate / low)	
Shape (irregular / lobular / oval /round)	
Margin (irregular / smooth / spiculated)	
**Dynamic contrast enhanced images**	
Tumour visible (y / n)	
Mass enhancement (y / n)	
MR assessment of focality (focal / multicentric / multifocal)	
Lymph node appearance (normal / abnormal, primarily guided by size)	
Shape (irregular / lobular / oval /round / non-mass-like enhancement)	
Location quadrant (lower inner, lower outer, upper inner, upper outer)	
Margin (irregular / smooth / spiculated / non-mass-like enhancement)	
**Diffusion-weighted images**	
Tumour visible (y / n)	

A radiographer (ZA), who had received specialised training for the task, delineated single-slice, 2-D ROIs on the slice corresponding to the maximal area of the largest lesion detected. These lesion outlines were confirmed (with modification where necessary) by an MR breast radiologist (EOF) with more than 5 years’ experience. Both the semantic features (categorical variables) and regions-of-interest were saved from ePAD to output files in the AIM XML format [25] and uploaded to the XNAT repository.

Approximately 20% of the classification subset (18 T2-weighted, 19 DCE and 19 DW-weighted images), evenly spaced throughout the data acquisition period and spread across scanner types, were recontoured by a senior radiologist (DMK) with more than 20 years’ experience, in order to assess inter-observer feature stability.

Annotators did not have access to any clinical data at the time of annotation and so were blinded to outcomes when assessing the pseudonymised images.

#### Radiomic feature calculation

In-house software, written in MATLAB (Mathworks, Natick, MA) and interfacing with XNAT for data access, was used to generate calculated radiomics features (Table 3) from the original DICOM files and the annotation AIM instance files. The project pre-dated the publication of the Image Biomarker Standardisation Initiative guidelines [2] and, hence, initial feature generation was performed with the aim of maintaining comparability with earlier studies. We implemented the feature set described by Aerts et al. [26]. This comprised 8 shape features, 14 first-order statistical features and 33 texture features calculated from the grey-level co-occurrence and run-length matrices. The original work by Aerts et al. also applies the first-order statistics and texture measures to 8 different wavelet decompositions of the original image data, to generate an additional set of 376 “wavelet” features. However, since a significant concern in this work was the relatively small number of patient datasets, we took the decision not to investigate the wavelet-based features described in [26] in order to minimise the potential for (a) model overfitting that might lead to overly optimistic estimates of performance; or, conversely, (b) introducing additional “noise” that might tend to reduce sensitivity, producing pessimistic assessments of algorithm performance.

**Table 3:** Calculated image region-of-interest features, as defined in the Supplementary Data of Aerts et al. [26]

**Shape features**	
Compactness 1	Spherical disproportion
Compactness 2	Surface area
Maximum diameter	Surface-to-volume ratio
Sphericity	Volume
**Statistical features**	
Energy	Minimum
Entropy	Range
Kurtosis	RMS
Maximum	Skewness
Mean	Standard deviation
Mean absolute deviation	Variance
Median	Uniformity
**Texture features**	
Autocorrelation	Inverse difference moment normalised
Cluster prominence	Inverse difference normalised
Cluster shade	Inverse variance
Cluster tendency	Long run emphasis
Contrast	Long run low grey level emphasis
Correlation	Low grey level run emphasis
Difference entropy	Maximum probability
Dissimilarity	Run length non-uniformity
Energy	Run percentage
Entropy	Short run emphasis
Grey level nonuniformity	Short run low grey level emphasis
High grey level run emphasis	Short run high grey level emphasis
Homogeneity 1	Sum average
Homogeneity 2	Sum entropy
Informational measure correlation 1	Sum variance
Informational measure correlation 2	Short run emphasis

#### Data normalisation

The effect of data normalisation has previously been examined by Schwier et al. [27], with equivocal results, and, based on these findings, it was decided not to normalise MR data for this study prior to feature calculation.

#### Clinical features

For each patient, clinical data relating to disease features and outcomes were extracted from clinical records, as shown in Table 4. Clinical variables were imported into R (R version 3.6.2, The R Foundation; RStudio version 1.2.5001, RStudio PBC) and underwent an initial curation process to convert the original free-text inputs into a smaller set of controlled terms, removing redundancy and correcting mis-annotations. The nodal status, established via pathological examination of lymph node sampling at surgery, was dichotomised as positive (which includes micrometastatic disease) or negative. Other categorical variables with more than two states were re-coded as binary one-hot factors. The following subset of clinical data from Table 4 (DCIS, IDC, ILC, LCIS, ER, PR, HER, molecular subtype, bilaterality, grade, clinically recorded tumour size) was then combined with the radiologist-defined semantic features of Table 2 and calculated radiomic features of Table 3 into a single R data frame for predictive and prognostic modelling.

**Table 4:** Patient demographics and clinical features available for evaluation

**Demographic data**	
Age	54 ± 12.5 [24–88], IQR 16.3
**Histological data**	
Receptor status ER (+/−)	negative (22), positive (133), NA (1)
Receptor status PR (+/−)	negative (44), positive (110), NA (2)
Receptor status HER2 (+/−)	negative (126), positive (16), NA (14)
Subtype	basal (12), HER2 (9), luminal (131), NA (4)
Molecular subtype	basal (12), HER2 (9), luminalA (113), luminalB (4), NA (18)
Grade	1 (11), 2 (102), 3 (41), NA (2)
Lymphovascular space invasion (LVSI) (determined at surgery)	absent (107), present (42), NA (7)
Nodal status (determined at surgery)	negative (99), positive (39), micrometastases (11), NA (7)
Disease pathology (multiple conditions allowed per patient)	DCIS (47), IDC (95), ILC (62), LCIS (23)
**Miscellaneous diagnostic data**	
Number of recorded lesions * For some patients, clinical measurements recorded after therapy noted no residual tumour	0* (7), 1 (133), 2 (14), 3 (2)
Laterality	left (87), right (63), bilateral (6)
Tumour size	(31 ± 24) [0–128] mm, IQR 26 mm
Focality (largest tumour)	unifocal (126), bifocal (12), multifocal (17), NA (1)
Type (largest tumour)	ductal (91), lobular (52), both (11), NA (2)
**Treatment**	
Surgery	Central excision (1), mastectomy (58), therapeutic mammoplasty (1), WLE (94), NA (2)
Neoadjuvant chemoendocrine therapy	no (112), yes (43), NA (1)
**Survival data**	
Date of diagnosis	March 2007 – July 2014
Date of imaging	January 2012 – December 2013
Date of surgery	January 2012 – September 2019
Date of death or last follow up	August 2013 – August 2019
Status at last follow-up	alive (140), dead (16)

### Statistical methods

#### Feature reduction

As a pre-processing step, inter-observer repeatability of feature values was assessed using the intraclass correlation coefficient (ICC) statistic with a two-way model and absolute agreement measure, denoted ICC(A, 1) in [28]. Features were rejected where the agreement between observers failed to reach the “good” threshold of ICC = 0.75 described in [29]. We next developed a novel correlation-based technique to remove redundant features prior to classification and time-to-event modelling. This approach uses the Spearman correlation coefficient and first removes correlated radiomic features independently *within* each of the three groups (shape, statistics and texture) using a standard algorithm (R findCorrelation() from the caret package with a cut-off value of 0.9). Then features that have *between*-group correlations greater than the same cut-off are removed, such that shape features are retained in preference to statistics or texture features, and statistics features are retained in preference to texture features. This has the effect of retaining features with simpler physical or statistical interpretations.

#### Model fitting

The data were used to inform on the following questions:

1. Can radiomics predict the presence of nodal metastases alone or in combination with clinical parameters?
2. Does radiomics improve survival modelling prediction either on its own or combined with clinical parameters?

#### Classification models

For the prediction of nodal metastasis, four different classification models were considered within R’s caret modelling framework: support vector machine [30], random forest [31], extreme gradient boosting [32] and naïve Bayes [33]. In all cases, we used caret’s built-in methods and ranges for tuning the model hyper-parameters, with the exception that for the XGBoost model we set the learning rate parameter eta to 0.0001. For the random forest model, we used 1000 trees, which was sufficient to ensure stochastic convergence of the classification performance estimates.

In addition to the unsupervised ICC and correlation feature reduction steps, recursive feature elimination (rfe, part of the caret package) was used as a supervised wrapper technique in combination with the previously mentioned classification models.

#### Validation of classification modelling

**Fig. 2:**
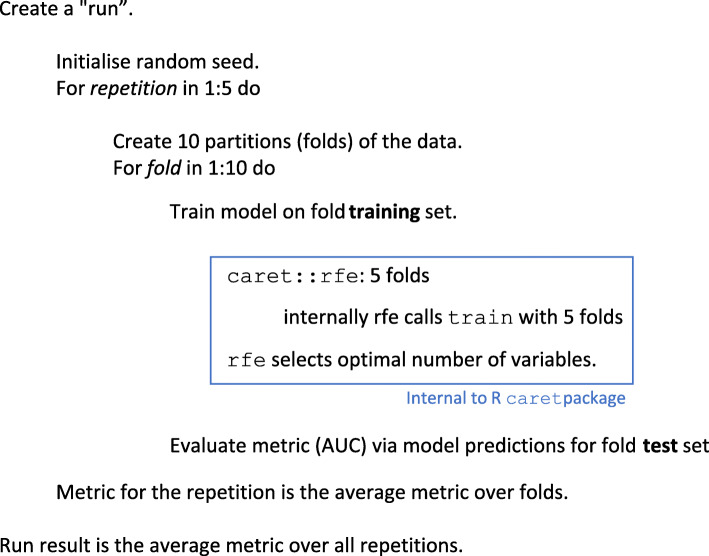
Pseudo-code describing model fitting, parameter tuning and performance estimating using a nested cross-validation process, as described in the text

Refs. [34, 35] provide useful information concerning different methods of validation. For our study, with limited numbers, we concluded that repeated cross-validation was the most appropriate methodology. The complete nested validation process is illustrated in Fig. 2 and includes 5-fold cross-validation internally within caret for the recursive feature elimination; 5-fold cross-validation for hyperparameter tuning; 10-fold cross-validation for classification performance estimation; and 5 repetitions to assess the variability of the overall procedure.

#### Performance metrics for classification

The output measure used to assess performance was the area under the receiver operator characteristic (ROC) curve (AUC), as calculated by the roc() and auc() functions of R’s pROC package. We examined in detail the modelling output and its distribution over the validation folds and repetitions. Variable importance plots were also created to aid interpretation of the models using the varImp() function of the caret package.

#### Survival modelling

For the time-to-event investigations we applied a random survival forest model [36] using the randomForestSRC package in R, with missing data imputation, the default tuning parameters and 20,000 trees to ensure convergence. Variable importance ranking was used to assess the relative impact of the features on the prediction performance. Overall performance was assessed using the out-of-bag prediction error, which is calculated as 1 – *C*, where *C* is Harrell’s concordance index [37], and for this metric lower values represent an improvement in predictive performance. The concordance index estimates the probability that, in a randomly selected pair of cases, the risk predicted by the model is higher for the case with the earlier event.

#### Missing clinical data

The classification models required that there were no missing data. This reduced the set of clinical parameters that could be used and/or the number of patients that could be included. (See Table 4 for details on the number of missing values for the clinical features.) We focused on the subset of patients for whom all clinical and imaging data were available, resulting in a *classification subset* with *n* = 92. By contrast, the random forest survival model used missing data imputation, which meant that we were able to include the majority of patients in a number of *survival subsets*, with exclusion only where radiomic data for particular image contrasts were unavailable.

## Results

### Prediction of nodal status

**Fig. 3:**
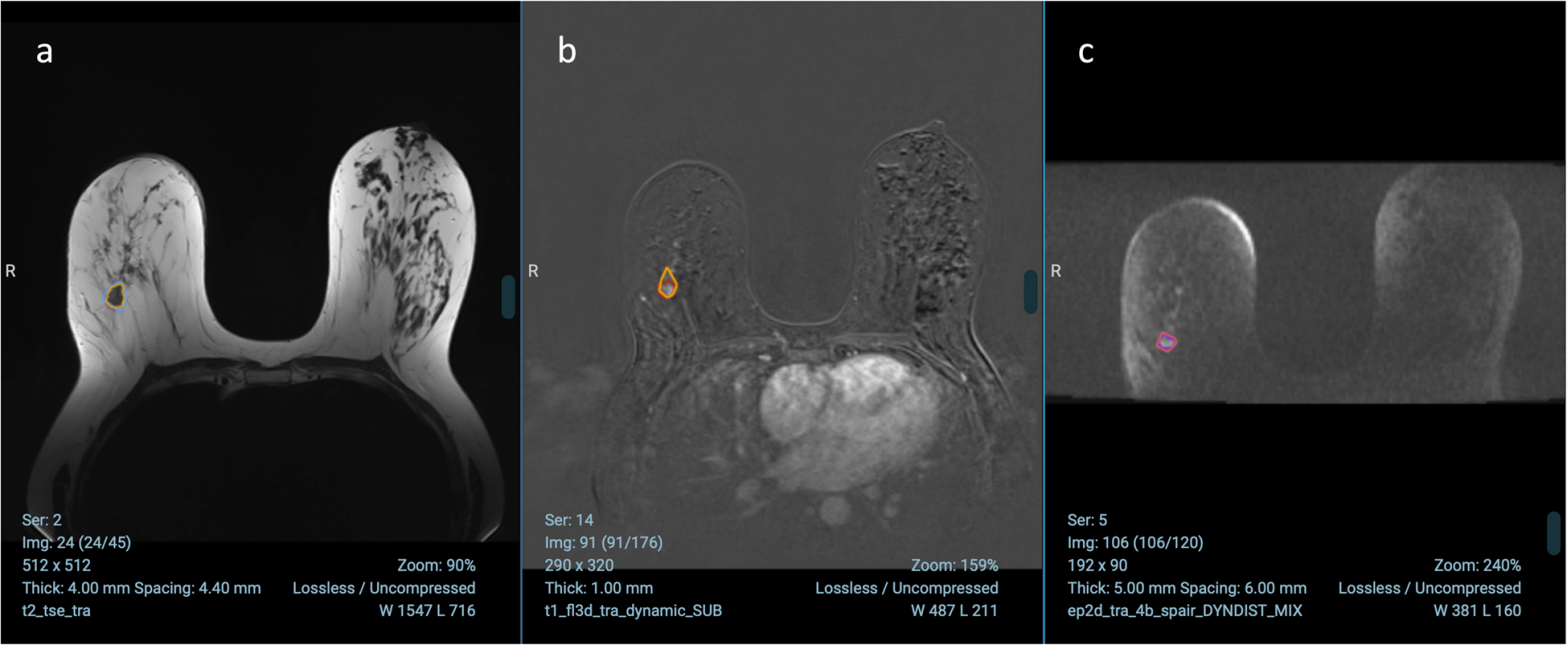
An exemplar image set showing both original ROIs and the repeat annotations for the ICC feature stability sub-study for (**a**) T2w-weighted (**b**) early-phase dynamic subtraction, and (**c**) diffusion-weighted images

**Fig. 4:**
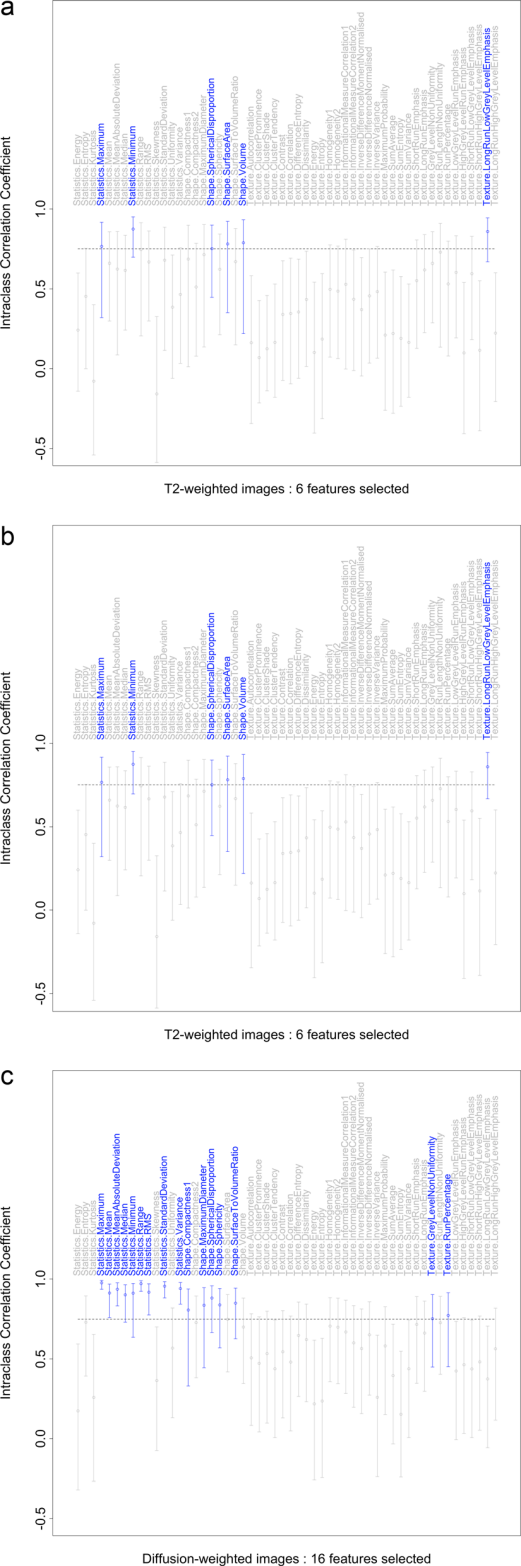
Radiomics features selected on the basis of the intraclass correlation coefficient (ICC), using a two-way “agreement” model, with threshold of 0.75, for the three different imaging contrasts

T2w, DCE and DWI image data for a typical subject are shown in Fig. 3, with both original and repeat regions of interest for the ICC analysis. Figure 4 illustrates our pre-processing to remove unstable features, with, respectively 6, 10 and 16 variables being retained for the three image contrasts.

**Fig. 5:**
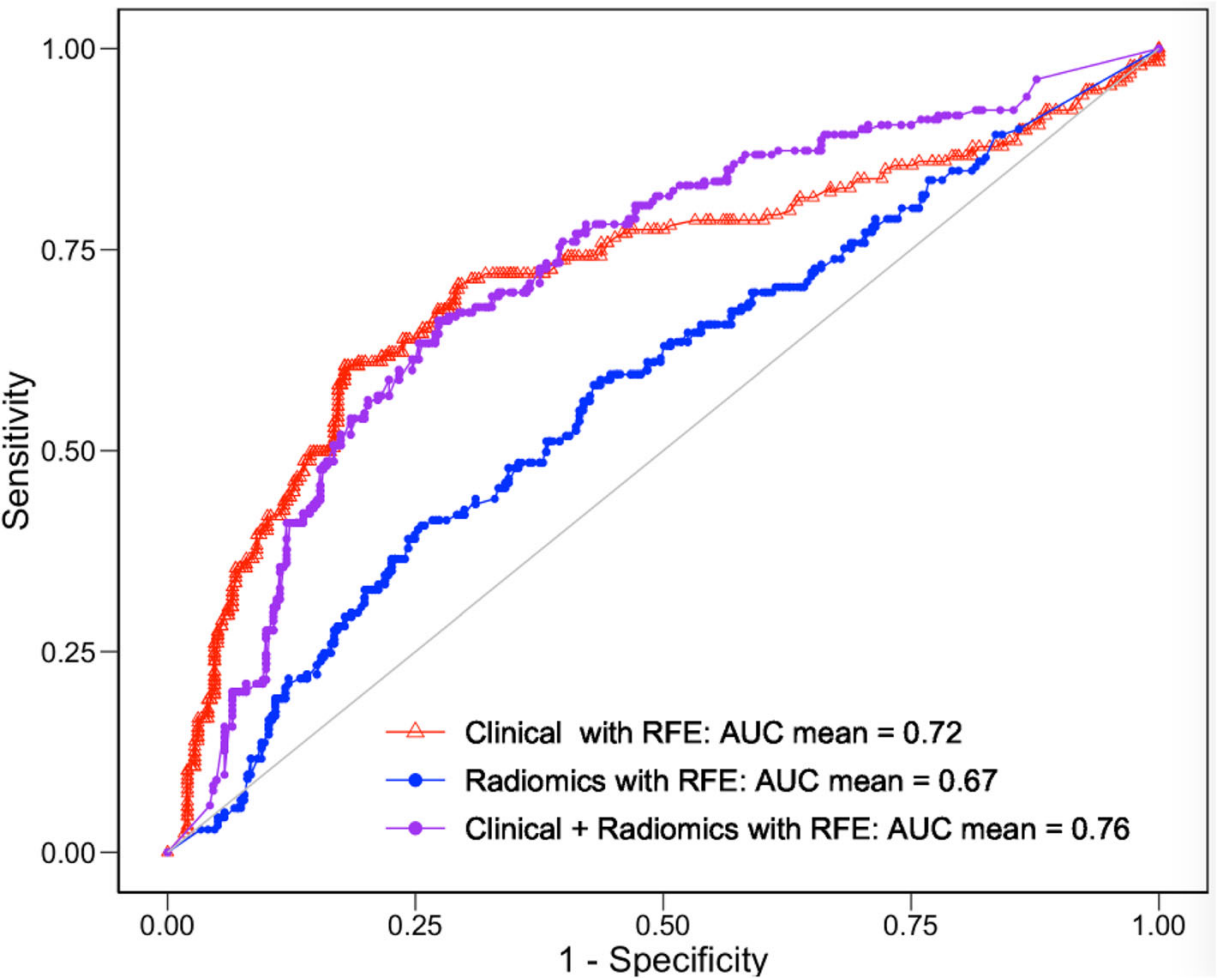
Mean ROC curves for nodal status classification problem using a Naïve Bayes classifier

**Fig. 6:**
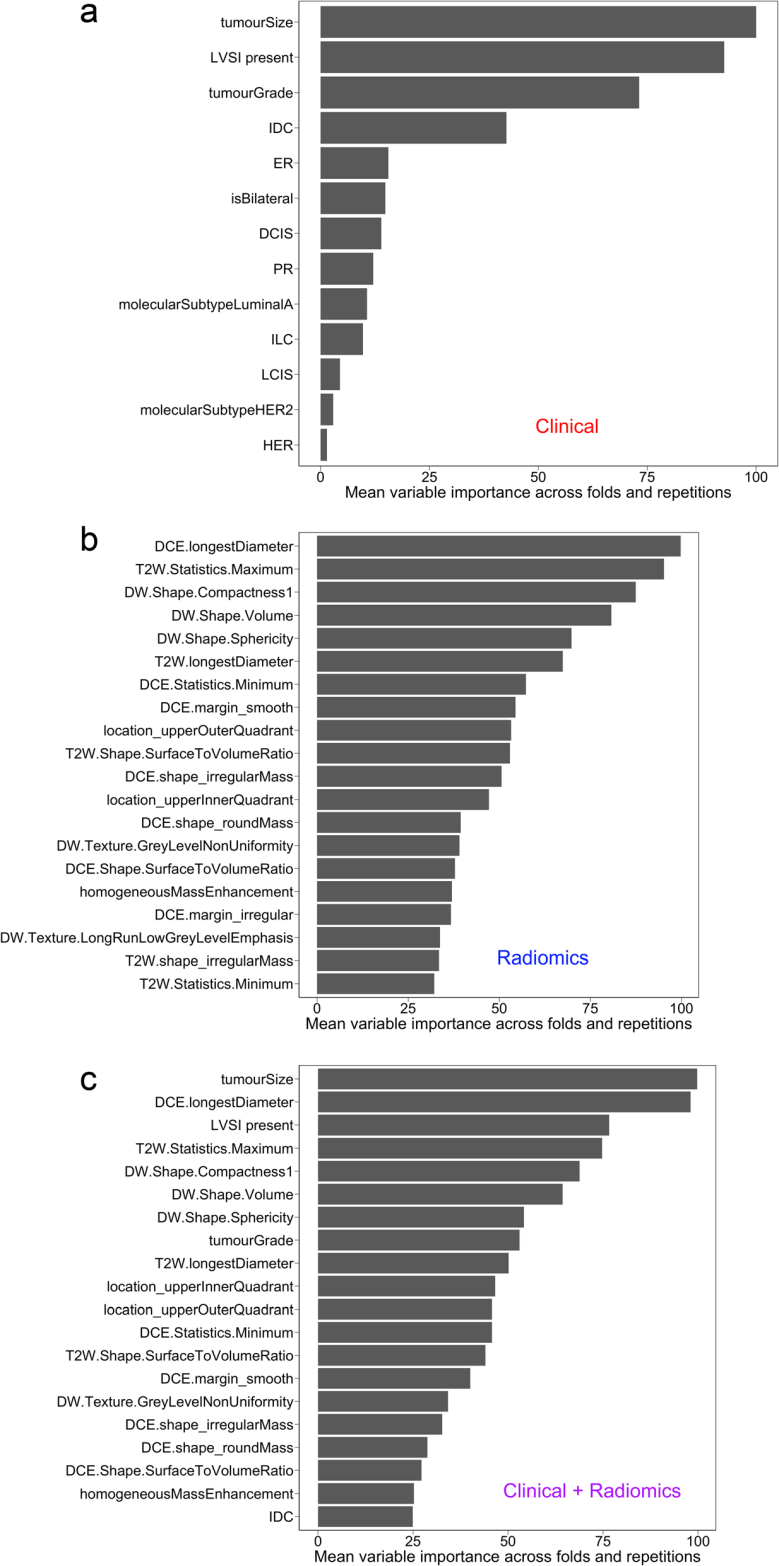
Analysis of the composition of models produced using recursive feature elimination: variable importance averaged across model folds and repetitions for models involving predictors drawn from (**a**) clinical data, (**b**) radiomics data (calculated plus semantic features, (**c**) clinical and radiomic data

Table 5 presents results for the four different model types studied. Figure 5 shows exemplar mean ROC curves for the *N* = 92 classification subset, using the naïve Bayes model applied to classify nodal status. Figure 5 demonstrates that clinical data alone (AUC = 0.735, SD 0.15) and radiomics features alone (AUC = 0.673, SD 0.16) are both predictive of nodal involvement at surgery. The combination of clinical and radiomics features resulted in a small improvement (AUC = 0.764, SD 0.14). Figure 6 presents the mean variable importance of the features contributing to the 50 separate models created (5 repetitions, 10 folds per repetition).

**Table 5:** Results of classification modelling for target variable lymph node status. Correlation-based feature selection (FS) refers to the method described in the Materials and Methods section, incorporating both ICC and Spearman rank correlations assessed in order of feature groups. Full feature selection starts with the features retained by the correlation-based approach and then applies R’s rfe algorithm under cross-validation. Results represent the mean AUC for 5 repetitions of 10-fold cross-validation, with standard deviations in the range 0.14–0.21 and standard error in the mean 0.02–0.03. However, the Individual data AUC values are not normally-distributed, independent random variables, and so these values should be regarded as indicative only and we do not quote an estimated confidence interval

Model type	Variables included	AUC (correlation-based FS)	AUC (correlation-based FS + recursive elimination)
SVM	Clinical	0.68	0.71
Random forest	Clinical	0.72	**0.74**
XGBoost	Clinical	0.68	0.72
Naïve Bayes	Clinical	0.71	0.72
SVM	Radiomics	0.55	0.62
Random forest	Radiomics	0.57	0.64
XGBoost	Radiomics	0.48	0.60
Naïve Bayes	Radiomics	0.65	**0.67**
SVM	Clinical + Radiomics	0.66	0.70
Random forest	Clinical + Radiomics	0.62	0.74
XGBoost	Clinical + Radiomics	0.56	0.71
Naïve Bayes	Clinical + Radiomics	0.67	**0.76**

### Confounding effects of scanner type

**Fig. 7:**
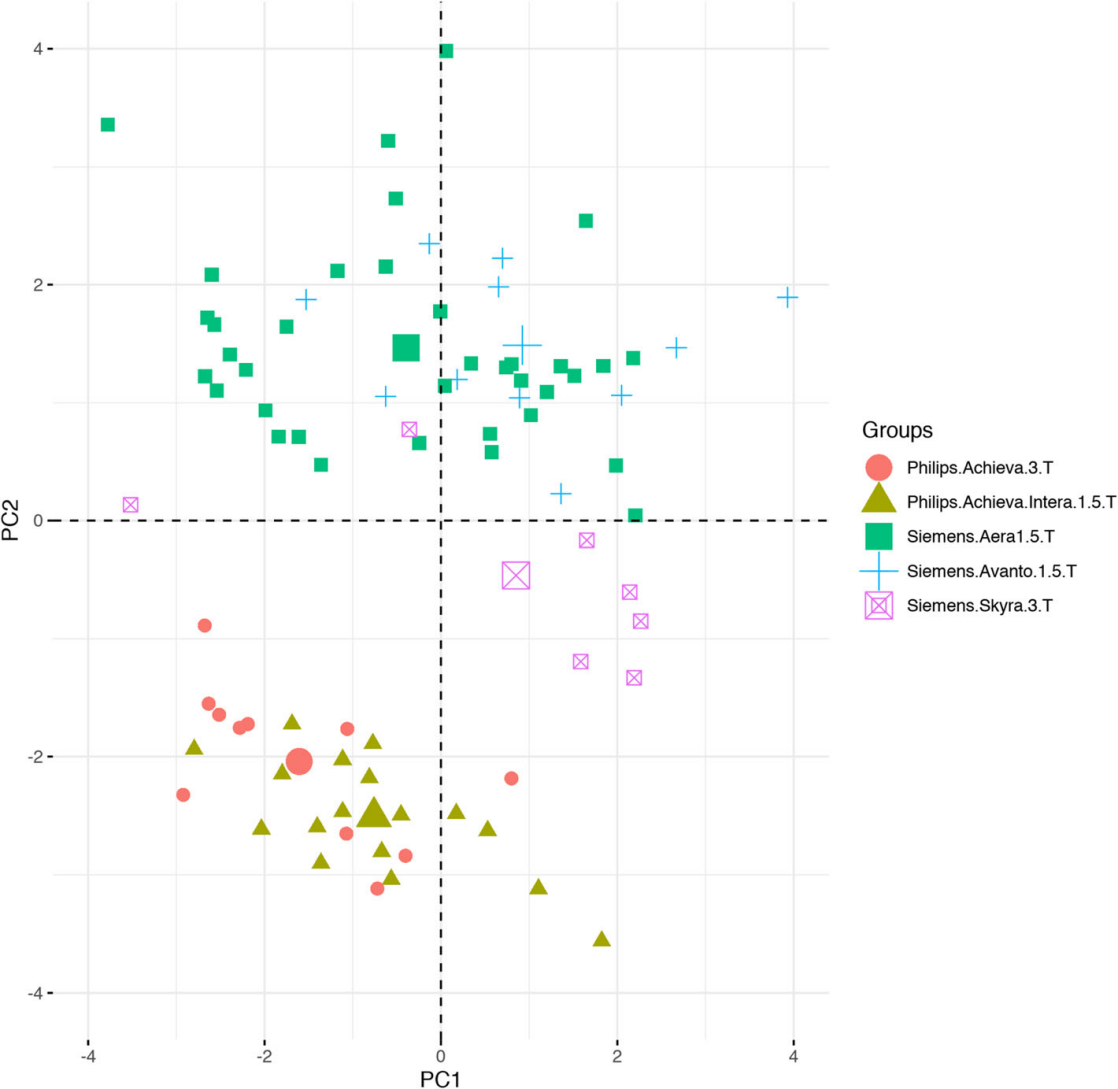
Principal component plot for the imaging feature data for all patients, with data points colour-coded by MR scanner type. Larger symbols represent group centroids. This partial separation via unsupervised classification methods demonstrates the significant extent to which data source acts as a confounding factor in radiomics studies of real-world data

**Fig. 8:**
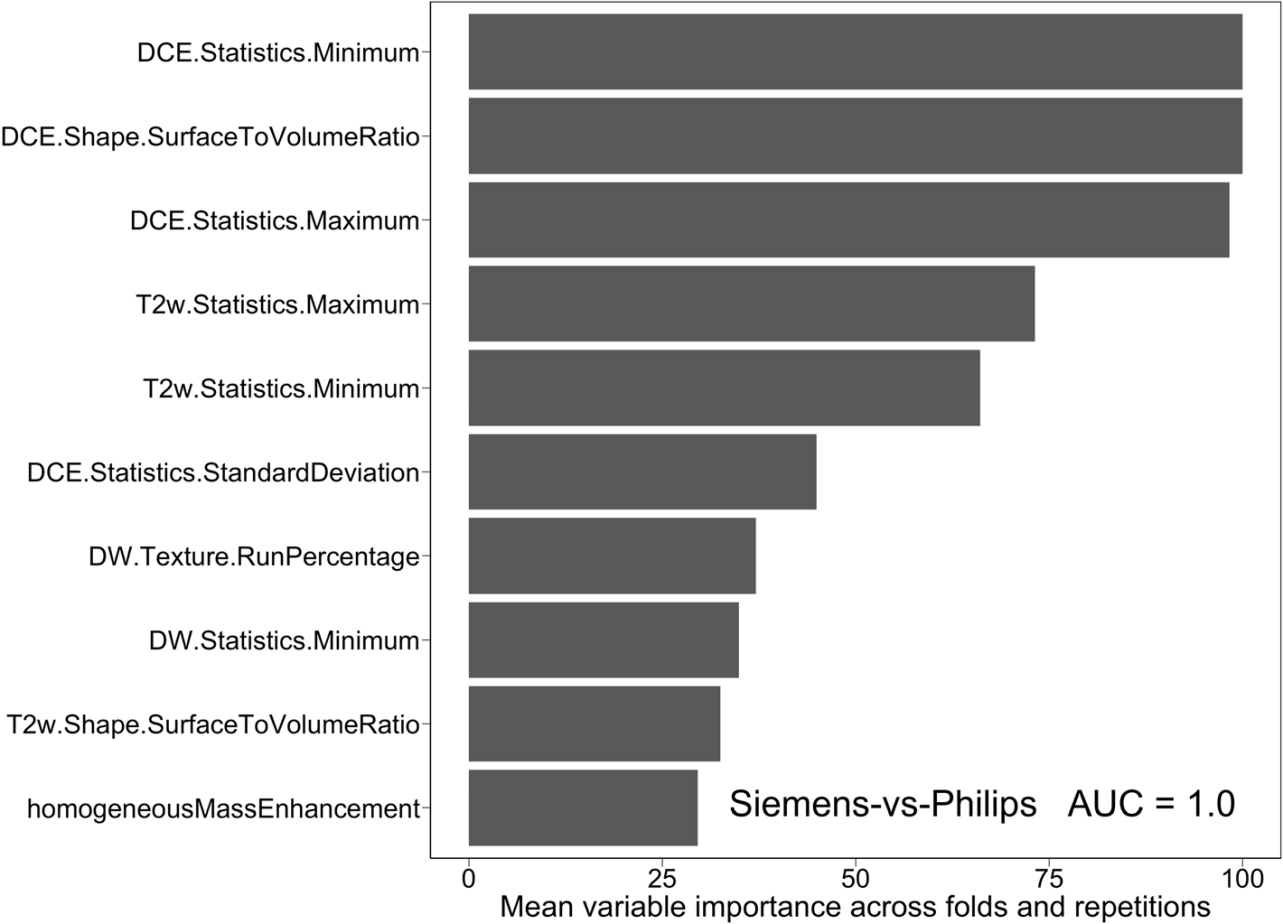
Mean variable importance for the top 10 variables in a Naïve Bayes fitted model to classify images by the manufacturer of scanner on which they were acquired, as an illustration of the degree to which scanner type is a confounding factor influencing the radiomics features

Figure 7 shows the result of a simple, unsupervised principal components analysis of the input data. Whilst separation of the data points is not complete, it is clear from this plot that scanner type is one of the important determinants in the two largest principal components and that points corresponding to data from scanners of the same type lie in similar regions of the diagram. Table 6 shows the results of a series of Naïve Bayes classification models, with the target as either scanner manufacturer, a specific scanner model or field strength. In all cases, either perfect (AUC = 1) or extremely good (AUC > 0.9) classification was achieved. As an exemplar, Fig. 8 displays the mean variable importance for the top 10 features returned in the final model for discriminating scanner manufacturer (Siemens vs. Philips).

**Table 6:** Results of classification modelling for the scanner related target variables. All classifications used only the calculated radiomic features, as passed to the models of Table 5 and there was no direct access by the models to either the raw pixel matrices or the DICOM header information

Classification target	AUC
Siemens Avanto 1.5 T vs. Rest	0.91
Siemens Aera 1.5 T vs. Rest	0.96
Philips Achieva 3 T vs. Rest	0.97
Philips Achieva or Intera 1.5 T vs. Rest	0.99
Siemens Skyra 3 T vs. Rest	0.97
1.5 T vs. 3 T	0.95
Siemens vs. Philips	1.00

### Prediction of disease survival

**Fig. 9:**
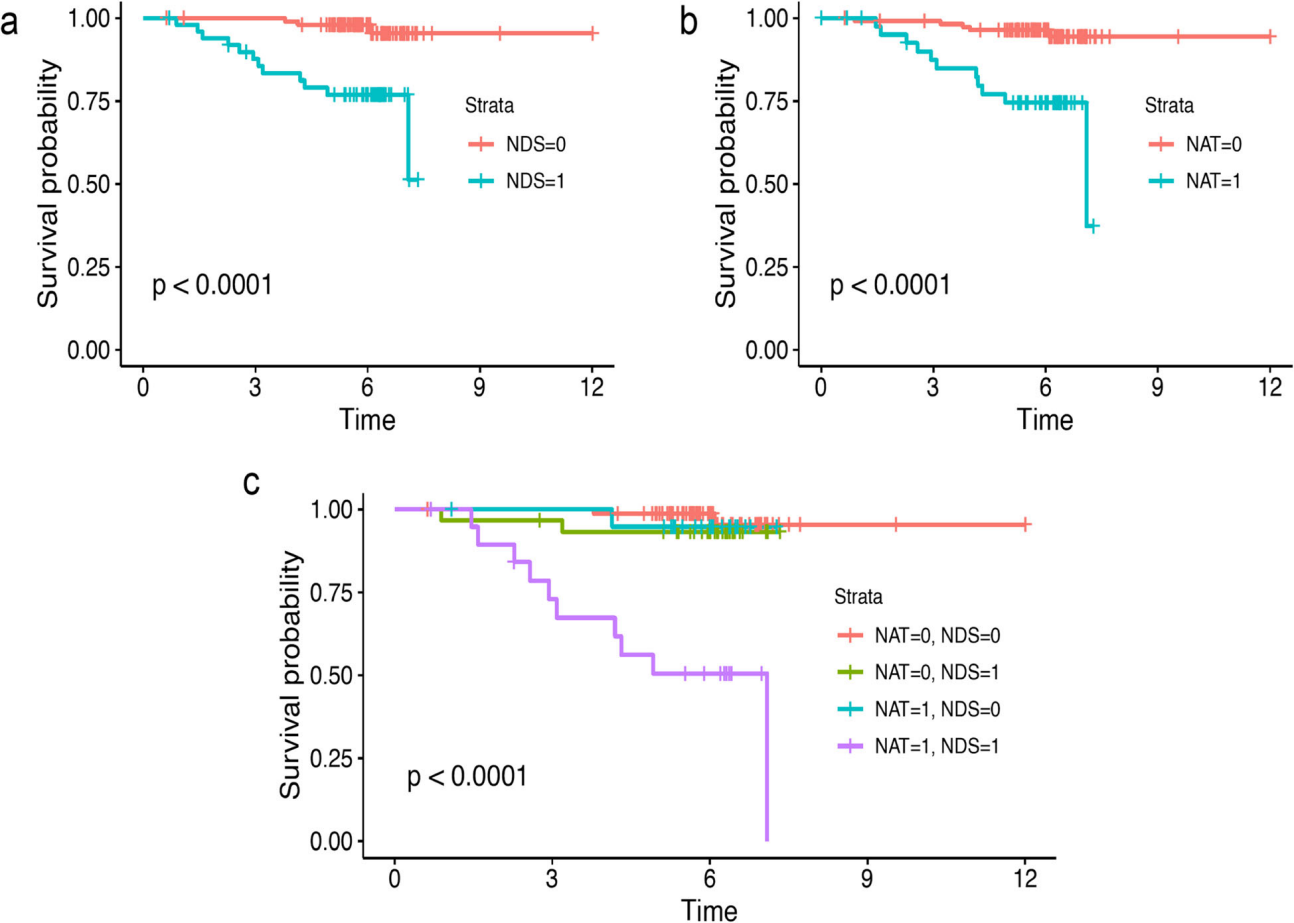
Kaplan-Meier plots for the survival data showing censoring events and separation of strata by: (**a**) nodal disease status (NDS); (**b**) tumour grade; (**c**) all combinations of nodal status and grade. Quoted *p*-values are for the null hypothesis that the survival curves for the given strata are the same. It will be seen that almost all death events come from the group that has nodal involvement and tumour grade 3

Table 7 presents the key findings of the survival analysis. The model based on clinicopathologic data alone performs moderately well, with an out-of-bag prediction error of 19.3%, and with the presence of nodal disease, tumour grade, and breast pathology (ILC or IDC), respectively, the clinical features with highest variable importance. The prediction error is worse for both models based on imaging features alone (36.9–51.8%) and combinations of imaging and clinicopathologic features (22.7–30.4%). Figure 9 shows Kaplan-Meier plots for the two most important clinicopathologic features (nodal disease and tumour grade) alone and in combination.

**Table 7:** Results of survival modelling

Feature groups considered	Number of subjects	Number of deaths	Prediction error (%)
Clinical	156	16	19.3
T2W radiomics	151	16	51.8
DCE radiomics	154	16	40.1
DW radiomics	135	15	36.9
Clinical +			
T2W radiomics	151	16	22.7
DCE radiomics	154	16	24.0
DW radiomics	135	15	25.5
Clinical + all radiomics	131	15	30.4

## Discussion

Obtaining radiomics results that are robust and can be generalised within and across institutions is currently a significant challenge for this new branch of data science. A key limitation of this and many other radiomics studies to date is access only to relatively small patient cohorts.

Two potential solutions exist. Firstly, one may seek to organise larger, prospective, multi-centre trials in order to obtain a critical mass of well-controlled data. In such cases, an adequate level of control may be obtained if all images are acquired with a common protocol, in the hope that a successful outcome will lead to a clinically adopted recommendation of a particular scanning methodology. An alternative controlling strategy would be to train models with datasets balanced between vendors, scanner models, field strengths, etc. in the hope that the corresponding radiomics models would be widely applicable. Which of these strategies is more effective is yet to be determined. However, in either case, for some questions, input data could take a significant time to accrue. Moreover, for rarer cancers, it may prove extremely difficult to organise well controlled imaging trials with sufficient power to test these methods prospectively.

The second approach is systematically to search large multi-hospital archives (for example, by natural language processing of radiology reports) for pre-existing patient data matching the relevant pathological and demographic criteria. This “real world” radiomics will, inevitably, assemble patient cohorts whose images have been acquired using different scanner types and imaging protocols. These are likely to depend on factors such as national guidelines, local business decisions, the original purpose of the scan and the generation of scanner (hence year of acquisition).

To a certain extent, incompatibilities between scans may be mitigated by pre-processing the images (for example, by suitable interpolation) in order to minimise “first-order” differences such as matrix size and spatial resolution. However, although significant progress has been made by the Imaging Biomarker Standardisation Initiative (IBSI) consortium [2] in developing reproducible methodologies, concerns remain that differences related to *data origin* in radiomics signatures may dominate more subtle effects associated with the analysis target. Failure of metrics to translate from one institution to another is thus often ascribed to differing conditions of image acquisition and/or the statistical “stability” of the radiomics features. Furthermore, whilst it seems intuitive that more discriminative radiomics signatures will be obtained from groups of patients all scanned with identical equipment and identical protocols, it is also highly probable that such signatures will perform best prospectively when new patients are scanned with these same protocols, and this limits their widespread applicability. Not only might the performance of these tests “in the wild” be uncertain, making regulatory approval problematic, but a methodology shown experimentally to work well might unexpectedly fail with the introduction of new equipment.

Hence, the purpose of this study was to investigate the extent to which a useful radiomic result could be obtained from heterogeneous input data, in particular different field strengths, scanner models and matrix sizes. This contrasts with the approach taken in previous studies aiming to predict lymph node status at surgery from diagnostic images [10–20], which typically involved images from a single vendor, model, field strength and imaging protocol. The hypothesis we wish to test here is *that some real effect (albeit possibly attenuated) is observable irrespective of the origin of the scan data and even in the presence of gross confounds in the input features.*

Figure 5 and Table 5 demonstrate that both clinical features and radiomics are separately prognostic of pathological nodal status. As might be expected, given the “noise” on the input data, the result using radiomics alone falls in the lower quartile of previously published AUC values and, in this case, would be unlikely to provide the basis for useful clinical test. Nevertheless, the combination of clinical and radiomics features is somewhat synergistic (AUC = 0.76) and in line with the six out of 12 prior publications that gave a comparable figure (0.63 [11], 0.87 [13], 0.87 [14], 0.84 [15], 0.82 [18] and 0.82 [21]).

Our methodology includes the important step of pre-selecting features based on the agreement between repeat annotation by different individuals. The low number of features retained for the T2w images reflects comments by the senior reviewing radiologist that the lesions showed little T2 contrast and outlines were difficult to draw. Notably, only three texture features remained overall after this step.

From Figs.ures 7 and 8 and Table 6, it is clear that the radiomic inputs, as expected, are highly predictive of the origin of the data and thus that they potentially present *confounds* in the input data for our modelling of lymph node status. Figure 7 suggests that *un*supervised methods of classification in these types of dataset are likely to highlight sources of difference between samples that are not the target of the analysis. Note that the image acquisition parameters themselves (e.g., matrix size) were not available to the model fitting, so no prima facie data leakage occurred that would allow algorithms to distinguish between models simply on the basis of relevant metadata within DICOM files. The Philips vs Siemens AUC = 1 of Fig. 8 may confirm anecdotal reports from radiologists that they are able to distinguish scanner manufacturer from the subjective appearance of the images, which might result from different vendor post-processing designs.

The fact that many high-scoring features are related to absolute intensities suggests scanner-specific image intensity scaling may be effective at reducing this confounder. However, MR image intensity is governed by a number factors other than vendor (e.g., proximity of target to receiver coil elements) and, moreover, is susceptible to both low- and high-intensity artefacts (e.g., motion artefacts in the subtraction images used in this study) and these could compromise any scaling based on intensity ranges in the input images. Further examination was beyond the scope of this paper, but the results of Schwier et al. [27] suggest that caution may be required.

Equally, other features such as surface-to-volume ratio could simply be proxies for a variable spatial resolution between scanners, an issue that might be soluble by careful adherence to IBSI protocols during feature generation. However, this remains to be tested.

Whilst our results are based on small numbers and relatively unbalanced classes in some cases, inspection of Fig. 8 (and equivalents for the other comparisons, data not shown) suggests that the discriminating radiomics features contain scanner-related information and it is these, rather than the characteristics of the tumour itself that play the dominant role in predicting scanner type. We note that several of the features from Fig. 8 are also present in Fig. 6b and c, potentially contaminating the radiomics signal and reducing the performance, but given their presence in the nodal status variable importance plots, it is also plausible that these features are influenced by both the tumour status and the image acquisition process.

Figure 6a shows that the most important clinical feature is tumour size. A linkage between size and the target variable is a relatively frequent finding in our experience of radiomics studies. All the remaining clinicopathological features classed as important are obtainable only via biopsy, or at the time of surgery in the case of lymphovascular space invasion (LVSI). Thus, if this and other recent work is confirmed by future, prospective studies, the ability of radiomics to make independent predictions at an earlier stage in the patient journey might be of clinical benefit.

Finally, it is interesting to note that the different image contrasts employed in the study all appear to be providing important information that is independent, with the top 10 features in the variable importance plot of Fig. 6c containing one image feature derived from the dynamic contrast data, two from the T2w data and three from the diffusion-weighted scans.

The survival data analysis part of the project produced more disappointing results, with radiomics being only weakly predictive (36.9–50.8% error rate, depending on the image contrast considered) compared with an 19.3% prediction error rate by clinical features alone, Table 6). Although the combination of clinical and radiomics features improved upon the result using radiomics alone, the error rate was still worse (22.7–30.4%) than for clinical data alone.

Three factors may explain this. Firstly, the overall number of events (deaths) in the survival analysis is low, making reliable estimation challenging. It was notable that all three of the models that include each of the T2W, DCE and DW radiomic features separately in combination with the clinical features give worse performance than the model for clinical features alone (Table 6). Similarly, the model that includes all radiomics features together with the clinical features is worse again than the three models just considered. Together, these findings suggest that when used to predict overall survival, the radiomics features (although slightly informative when considered alone – see Table 6) are effectively a noisy signal that, given the low number of events, the model is unable to filter successfully.

Secondly, as can be seen in Fig. 9, the censoring pattern is highly concentrated, with 90% of the censored events occurring being between 5 and 8 years, corresponding to a time when patients will have stopped regular follow-up. This means that the variability of the input features observed between these censored patients contains very little information that the model can use to predict differences in survival time. Inference of the connection between the input features and survival time is, then, mainly driven by the non-censored patients, and there is a low number of observed events in this study.

Thirdly, variable importance analysis of the clinical-only data indicates that the two top-ranked prognostic features of the clinical data are the presence of nodal disease at surgery, and tumour grade. Figure 7 shows Kaplan-Meier curves for each of these features separately and in combination, where it is apparent that, when taken alone, the nodal status (Fig. 9a) and the tumour grade (Fig. 9b) each show a degree of risk stratification separately, but when taken together they give clear stratification with the high-risk group (Fig. 9c, pink curve corresponding to patients with nodal disease and tumour grade 3) predominantly consisting of patients who died, with the other three groups dominated by censored patients. To improve the risk stratification any further by adding additional features (in particular radiomics features), any additional features would need to contain information to explain the survival time differences between patients in the high-risk group, which as previously mentioned, are insufficient in number to support such inference.

## Conclusions

In this paper, we have articulated the reasons why what we have called “real world” radiomics is an important subject to study. Almost inevitably, AI tools of the future will need to be applied to heterogeneous data from different manufacturers, scanner types and MR field strengths. We have examined one such example, the prediction of lymph node status, and found that a useful, common radiomic signature can be obtained from patients scanned on six different scanners installed in our institution. We have also confirmed the ability of the radiomic data to predict scanner model, manufacturer and field strength, but have shown that, whilst this information is clearly present in the radiomic features and acts as a confound for unsupervised classification, the types of supervised classification algorithm now commonly used in radiomics are able to overcome these distractors and create models that successfully predict the target variable. For this limited study, we found that our radiomics features gave poorer predictions of survival than clinicopathologic ones and have discussed potential reasons for this.

Further investigation of these effects in other pathologies and with larger patient cohorts would be of significant interest.

## Supplementary Information

**Additional file 1.**

**Additional file 2.**

## Data Availability

The datasets analysed during the current study are not publicly available for information governance reasons. Requests for access will be reviewed on an individual basis on application to the corresponding author.

## Abbreviations

AIM: Annotation and Image Markup
AUC: Area Under the Curve
BI-RADS: Breast Imaging-Reporting and Data System
CI: Confidence Interval
CT: Computed Tomography
DCE: Dynamic Contrast-Enhanced
DCIS: Ductal Carcinoma In Situ
DICOM: Digital Imaging and Communications in Medicine
DW: Diffusion Weighted
ER: Estrogen Receptor
HER: Human Epidermal Growth Factor Receptor
IBSI: Image Biomarker Standardisation Initiative
IDC: Invasive Ductal Carcinoma
ILC: Invasive Lobular Carcinoma
LCIS: Lobular Carcinoma In Situ
LVSI: Lymphovascular Space Invasion
MRI: Magnetic Resonance Imaging
PET: Positron Emission Tomography
PR: Progesterone Receptor
ROC: Receiver Operator Characteristic
T1W: T1-weighted
T2W: T2-weighted
VIMP: Variable Importance
XNAT: eXtensible Neuroimaging Archive Toolkit
